# First demonstration of coherent Cherenkov radiation matched to circular plane wave

**DOI:** 10.1038/s41598-017-17822-z

**Published:** 2017-12-12

**Authors:** Norihiro Sei, Toshiharu Takahashi

**Affiliations:** 1 Research Institute for Measurement and Analytical Instrumentation, National Institute of Advanced Industrial Science and Technology, 1-1-1 Umezono, Tsukuba, Ibaraki 305-8568 Japan; 2 Research Reactor Institute, Kyoto University, 2 Asashiro-nishi, Kumatori, Osaka 590-0494 Japan

## Abstract

We observed coherent Cherenkov radiation matched to a circular plane wave (CCR-MCP) for the first time using a hollow conical dielectric made of a high-density polyethylene. The refractive index and the absorption coefficient of the dielectric were evaluated to be 1.537 ± 0.004 and 0.006 ± 0.028 by measuring the pulse formed by the interference between the CCR-MCP and the coherent diffraction radiation. These values were consistent with the values shown in a reference for the high-density polyethylene. In accordance with the theory of the Cherenkov radiation, the spectrum of the CCR-MCP shifted towards higher wavenumbers compared to that of the coherent diffraction radiation. The intensity of the CCR-MCP beam was proportional to the height of the hollow conical dielectric and was 3 times the intensity of the coherent diffraction radiation. The CCR-MCP technique can produce broadband terahertz-wave sources with unprecedented power at compact accelerator facilities.

## Introduction

The terahertz (THz) region of an electromagnetic spectrum has been actively studied in non-linear optics. Several techniques utilizing the THz region have been pioneered, such as multiple exciton generation under strong electric fields and ablation dynamics of molecules^1–3^. These studies are technologically supported by intense THz wave sources used together with short-pulse lasers. Especially notable is a technique of tilted-pulse-front excitation that can develop THz wave sources with pulse energies exceeding 10 μJ^4,5^.

Electron accelerators have also been used to generate intense wave sources in the THz region^6–12^. Accelerator-based wave sources have an advantage in that the repetition frequency is high. However, a large accelerator facility is necessary to develop a broadband THz wave source with a pulse energy on the order of μJ. In order to obtain such an intense wave source in an existing compact accelerator facility, a new method of generating the wave source should be developed that may be used instead of the coherent synchrotron radiation and coherent transition radiation (CTR)^7,13^. Recently, we proposed a method of coherent Cherenkov radiation (CCR) matched to a circular plane wave that utilized a hollow conical dielectric^14^. Because the electron beam passes through the hollow part of the conical dielectric to generate the CCR beam^15^, the loss of the electron beam is negligible. When the angle between the generatrix and the rotation axis is half of the angle of the CCR, generated on the inner surface of the hollow conical dielectric, the CCR is entirely reflected from the conical surface and the radiation phase is matched  on the basal plane. Using a transparent material in the THz region as the dielectric, the intensity of the CCR beam becomes proportional to the height of the hollow conical dielectric and much higher than that of the CTR. We conducted experiments to observe the CCR matched to a circular plane wave (CCR-MCP) using an L-band linac at the Kyoto University Research Reactor Institute (KURRI-LINAC)^16^. In this article, we describe the successful formation of the CCR-MCP beam and report its measured properties. We show that CCR-MCP can be a powerful THz-wave source in the existing compact accelerator facilities.

## Results

### Observation of the CCR-MCP

**Figure 1:**
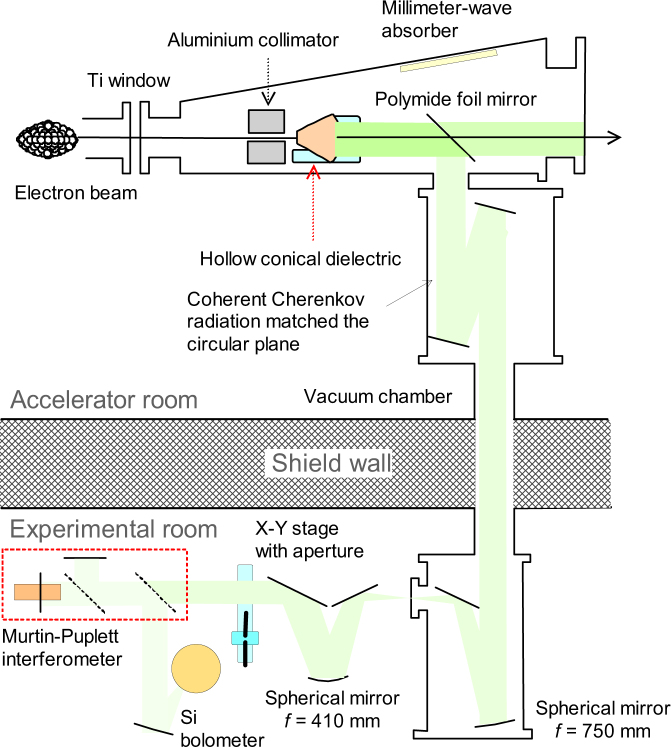
Schematic layout of the CCR-MCP experiments at the Kyoto University Research Reactor Institute.

The schematic layout of the CCR-MCP experiments is shown in Fig. 1. In the CCR-MCP experiments, a hollow conical dielectric was inserted at a position of 0.4 m in front of an aluminum-foil mirror, which was used in ordinary CTR experiments at the KURRI-LINAC^17–20^. The hollow conical dielectric was made of a high-density polyethylene (HDPE) with the inner diameter of 10 mm. According to the ref.^21^ the refractive index of the HDPE is 1.53 in the sub-THz region. The CCR is generated on the inner surface of the hollow conical dielectric. In order to match the phase of the CCR on the basal plane, the angle between the generatrix and the rotation axis was set to 24.8 degrees, which was half of the Cherenkov angle of the HDPE. Three kinds of the hollow conical dielectrics with heights of 40, 60 and 80 mm were prepared. Because the root-mean-square size of the electron beam was as large as 3 mm or more^22^, an aluminium collimator was positioned 50 mm in front of the hollow conical dielectric. The collimator was 150 mm in length with the inner diameter of 8 mm. The CCR beam emitted from the basal plane was separated from the electron beam by a polyimide film with the thickness of 50 μm, which was installed instead of the aluminium-foil mirror. Although the reflectance of the polyimide is 8% in the sub-THz region^23^, the electron beam does not generate CTR on it. The CCR beam reflected by the polyimide film was transported to the experimental room using the flat mirrors. It was focused by a spherical concave mirror with the focal length of 0.75 m and subsequently, converted into a parallel beam by a spherical concave mirror with the focal length of 0.41 m. The distance, *L*_*d*_, between the hollow conical dielectric and the upstream spherical concave mirror in the experimental room was 9.5 m. The CCR beam was reflected in the horizontal plane, and the incident angle on the short-focal spherical concave mirror was 17 degrees.

**Figure 2:**
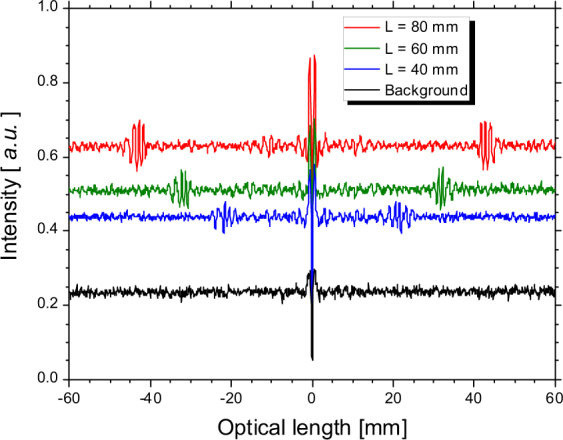
Measured interferograms due to the background radiation (black line) and due to the hollow conical dielectrics with the height of 40 (blue line), 60 (green line) and 80 mm (red line), using the Martin-Puplett-type interferometer.

The intensity of the CCR emitted by an electron beam away from a dielectric increases as the energy of the electron increases^24^. Therefore, the energy of our electron beam was set to 40 MeV. With the aluminium collimator, the charge in a micropulse passing the hollow conical dielectric was 77 pC and the electron-beam current was approximately 30 nA. The silicon bolometer detected not only the CCR-MCP but also the sub-THz-wave background radiation that was generated by the electron beam in the vacuum chamber. The main component of the background radiation was the coherent diffraction radiation (CDR) generated at the exit of the aluminium collimator^25^. Because the charge in a micropulse was low due to the collimator, the energy of the CDR per 10 ns macropulse was approximately 0.5 nJ. The background radiation could be measured by rotating the hollow conical dielectric by 180 degrees so that the face of the basal plane is directed towards the incident direction of the electron beam. The measured interferograms for the hollow conical dielectrics with the heights of 40, 60, and 80 mm and for the background radiation are shown in Fig. 2. Note that there are satellite pulses, which are interference waves between the CCR and the CDR, in the interferograms for the hollow conical dielectrics. Because the CCR passed through the dielectric, the CCR pulse was detected later than the CDR pulse. The distance of the satellite pulse from the centre burst position was proportional to the height of the hollow conical dielectric *L*_*h*_^14^. This distance Δ is given by the following equation:

1 $${\rm{\Delta }}=(n-1){L}_{h},$$

where *n* is the refractive index of the dielectric. By fitting the data for Δ and *L*_*h*_ obtained from Fig. 2 to equation (1), *n* was evaluated to be 1.537 ± 0.004. This value was in good agreement with the refractive index of the HDPE provided in ref.^21^ Although the width of the satellite pulse did not depend on *L*_*h*_, the height of the satellite pulse increased as the *L*_*h*_ increased. The base line of the interferogram denotes the intensity of the radiation. The intensity of the CCR-MCP can be evaluated by calculating the difference from the relative intensity of the background radiation. According to the theory of the CCR-MCP, the intensity is related to *L*_*h*_ by the following equation^14^:

2 $$P\propto \frac{1}{{\alpha }_{d}}[\exp (-\frac{n}{n+1}{\alpha }_{d}{L}_{h})-\exp (-{\alpha }_{d}{L}_{h})],$$

where *α*_*d*_ is an absorption coefficient of the hollow conical dielectric. By fitting the data of the intensity of the CCR-MCP and *L*_*h*_ obtained from Fig. 2 to equation (2), *α*_*d*_ was evaluated to be 0.006 ± 0.028. We note that the HDPE hardly absorbs any sub-THz waves and that the calculated value is consistent with the absorption coefficient shown in ref.^21^

**Figure 3:**
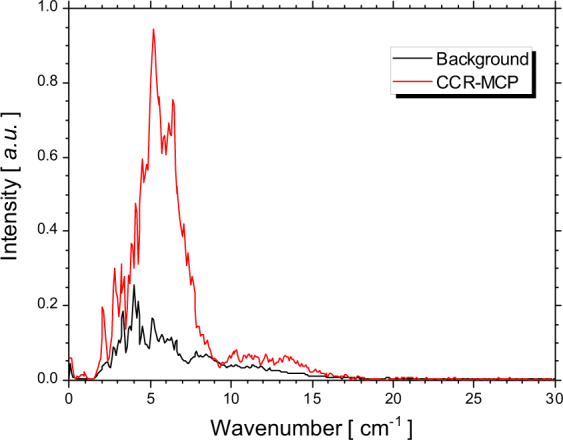
Measured spectra of the background radiation (black line) and the CCR-MCP using the hollow conical dielectric with the height of 80 mm (red line).

**Figure 4:**
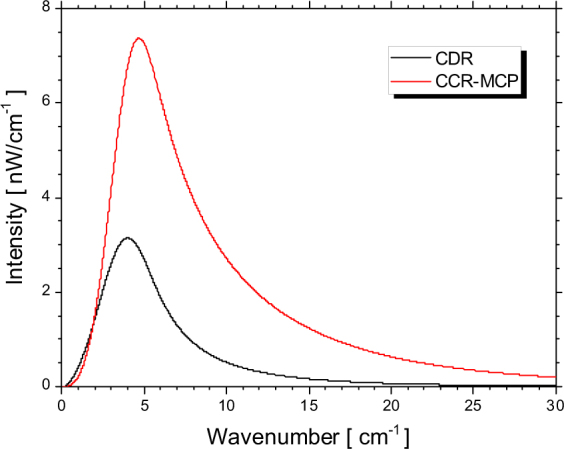
Calculated spectra of the CDR (black line) and the CCR-MCP using the hollow conical dielectric with the height of 80 mm (red line).

The spectrum of the CCR-MCP can be evaluated for the measured interferogram by using a fast Fourier transform. Figure 3 shows the spectra calculated for the CCR-MCP of the hollow conical dielectric with the height of 80 mm and for the background radiation. In these spectral measurements, the electron-beam current was 43 nA. We note that the CCR-MCP had higher intensity compared to the transported CDR and its spectrum shifted towards higher wavenumbers. To further investigate these spectral characteristics, we estimated the radiation spectra of the electron bunch uniformly distributed over a distance of 14 mm^13^. In the case of the CDR, the form factor *f*(*λ*) is almost equal to the longitudinal form factor *f*_*L*_(*λ*) given by the following equation^26^:

3 $$f(\lambda )\cong {f}_{L}(\lambda )={\{\frac{\sin (\frac{2\pi {\sigma }_{L}}{\lambda })}{\frac{2\pi {\sigma }_{L}}{\lambda }}\}}^{2},$$

where *λ* is the wavelength of the CDR and 2*σ*_*L*_ is the longitudinal bunch length of the electron beam. Because *f*_*L*_(*λ*) is an oscillating function, we use an envelope of *f*_*L*_(*λ*) as the form factor for the CDR. Furthermore, the Cherenkov radiation is emitted at a large angle from the electron beam axis, while the intensity of the CCR is influenced by the transverse size of the electron beam^24^. In the case of CCR-MCP, however, the whole radiation is emitted in the direction of the electron beam. By focusing the CCR-MCP beam emitted from the basal plane of the hollow conical dielectric, without disturbing its phase, the intensity of the CCR-MCP becomes dependent only on the longitudinal structure of the electron bunch. We further assume that the form factor for the CCR-MCP is equal to the form factor for the CDR. Figure 4 shows the calculated spectra of the CDR and the CCR-MCP with the hollow conical dielectric that is 80 mm in height. These calculations indicate that the intensity of the CCR-MCP is 3 times the intensity of the CDR. The maximum of the calculated spectra was at 4.0 cm^−1^ for the CDR and at 4.7 cm^−1^ for the CCR-MCP. On the other hand, the maximum of the measured spectra from Fig. 3 was at 4.0 cm^−1^ for the CDR and at 5.2 cm^−1^ for the CCR-MCP. In this case, the CCR-MCP spectrum also shifted towards higher wavenumbers compared to the CDR spectrum. The reason is that the intensity of diffraction radiation is almost independent of the frequency in the sub-THz region while the intensity of Cherenkov radiation increases in proportion to the frequency^27^. Compared to the calculated spectra, the measured spectra lacked in the higher wavenumber region. In order to reproduce the measured spectra by the simulations, a precise structure of the electron bunch is necessary. However, we first achieved the observation of the CCR-MCP which was more powerful than the CDR. And it was demonstrated that the spectrum of the CCR-MCP shifted to the higher wavenumber side than that of the CDR.

### Measurement of the CCR-MCP profile

**Figure 5:**
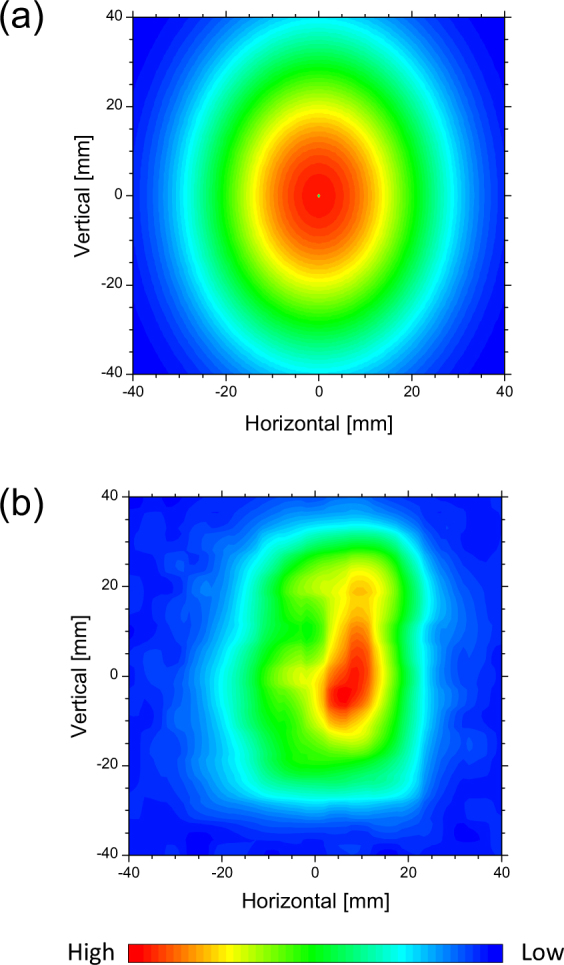
Calculated (**a**) and measured (**b**) two-dimensional distributions of the CCR-MCP using the hollow conical dielectric with the height of 80 mm at a position 0.6 m away from the downstream spherical concave mirror.

Because the CCR-MCP uses a hollow conical dielectric, the two-dimensional distribution of the CCR beam on the basal plane of the dielectric also has a hollow structure. However, the distance *L*_*d*_ is much larger than the wavelength of the CCR-MCP at the KURRI-LINAC, consequently, the hollow structure cannot be maintained due to the diffraction effect in the experimental room. Because the wavelength *λ*_*C*_ of the CCR-MCP spectrum maximum is approximately 2 mm, the following condition holds in the experimental room:

4 $${L}_{d} >  > \frac{{D}^{2}}{{\lambda }_{C}},$$

where *D* is the radius of the basal plane of the hollow conical dielectric. Therefore, the two-dimensional distribution of the CCR-MCP beam in the experimental room can be calculated with the Fraunhofer diffraction approximation. Figure 5(a) shows the two-dimensional distribution of the CCR-MCP beam calculated for the hollow conical dielectric with the height of 80 mm, positioned 0.6 m away from the downstream spherical concave mirror. Because the short-focus concave mirror is used instead of a toroidal mirror to make a parallel beam, the profile of the CCR-MCP beam is an ellipse with the short axis in the horizontal direction. The standard deviation of the calculated two-dimensional distribution is 18 mm in the horizontal direction and 24 mm in the vertical direction.

We measured the two-dimensional distribution of the CCR-MCP beam in the experimental room. An aperture with a diameter of 10 mm, which was set on an *X*–*Y* axis translation stage having an area of 80–80 mm^2^, was positioned 0.6 m from the downstream spherical concave mirror. The radiation power that passed through the aperture was measured by the silicon bolometer in 5-mm steps in the horizontal and vertical directions. Figure 5(b) shows the measured two-dimensional distribution of the CCR-MCP beam using the hollow conical dielectric with the height of 80 mm. The standard deviations of the measured two-dimensional distribution was 14 ± 1 mm in the horizontal direction and 20 ± 1 mm in the vertical direction. The measured standard deviations are about 20% smaller than the calculated ones. However, we note that both the measured profile and the calculated profile have a similar, shorter axis in the horizontal direction. In order to observe the two-dimensional distribution of the hollow structure that is specific to the CCR-MCP beam, it is necessary to make paraxial approximation. Because the bunch length of the electron beam in the KURRI-LINAC is not short, it is difficult to make the wavelength of the coherent radiation shorter. In future experiments, we plan to measure the two-dimensional distribution of the CCR-MCP beam when the aperture is positioned near the hollow conical dielectric.

## Discussion

We observed the CCR-MCP for the first time using the KURRI-LINAC in Kyoto University. The CCR emitted by the hollow conical dielectric, made of the HDPE, was delayed compared to the CDR emitted by the aluminium collimator. The delay time was proportional to the height of the hollow conical dielectric. The refractive index of the HDPE evaluated from the delay time was in good agreement with the result found in ref.^21^ Moreover, the absorption coefficient of the HDPE evaluated from the intensity of the CCR was also consistent with the result provided in ref.^21^ According to the theory of the CCR-MCP, the spectrum of the CCR beam measured with the Martin-Puplett-type interferometer should be shifted towards shorter wavelengths compared to that of the CDR. The beam profile measured in the experimental room generally agreed with the two-dimensional distribution calculated using the Fraunhofer diffraction approximation. These experimental results support our conclusion that the observed radiation was, in fact, CCR-MCP beam.

We demonstrated that the CCR-MCP had higher intensity than the CDR. By using an electron beam with a smaller beam size, the inner diameter of the hollow conical dielectric can be reduced. This would enable one to generate the CCR-MCP beam with a shorter wavelength and a higher intensity. We plan to develop an intense wave source based on the CCR-MCP at the Laboratory for Electron Beam Research and Application (LEBRA) in Nihon University, where previously a CTR with the energy of 0.1 μJ per micropulse has been developed^24,28^. Because the bunch length of the electron beam with the charge of 30 pC per micropulse can be made shorter than 0.5 ps at the LEBRA, creating a broadband THz-wave source with the energy of 1 μJ per micropulse or more will become possible^14^. By using a higher-quality electron beam, it is not difficult to develop a broadband THz-wave source with the pulse energy exceeding 10 μJ. The CCR-MCP technique enable one to produce intense THz-wave sources at compact accelerator facilities.

## Methods

The CCR-MCP experiments were performed at the KURRI-LINAC, where studies of various types of coherent radiation have been conducted and a beamline for millimetre-wave and THz-wave spectroscopy has been constructed using the CTR^17,18^. The electron beam can be accelerated up to approximately 40 MeV and the charge in a micropulse of the electron beam can be greater than 1 nC. The accelerator tubes are operated at the frequency of 1.3 GHz so that the interval between the micropulses is 230.5 mm. The macropulse duration of the electron beam is adjustable from 2 to 100 ns in the short-pulse mode. The electron beam is injected into a vacuum chamber from the accelerator via a thin titanium window. The CTR is generated by the electron beam at the titanium window and at the aluminum-foil mirror in the vacuum chamber^19^. The CTR is then focused into a parallel beam with a concave mirror having a focal distance of 1.5 m. The beam is subsequently transported in a vacuum and extracted to the air through a Mylar window in the experimental room. It is then injected into a Martin-Puplett-type interferometer with a silicon bolometer for spectroscopy experiments^20^.
